# Prevalence of hypogammaglobulinemia and its management with subcutaneous immunoglobulin supplementation in patients after allogeneic hematopoietic stem cell transplantation—a single-center analysis

**DOI:** 10.1007/s00277-021-04649-y

**Published:** 2021-09-03

**Authors:** Ewa Karakulska-Prystupiuk, Jadwiga Dwilewicz-Trojaczek, Joanna Drozd-Sokołowska, Ewelina Kmin, Marcin Chlebus, Karolina Szczypińska, Piotr Boguradzki, Agnieszka Tomaszewska, Krzysztof Mądry, Jarosław Biliński, Grzegorz Władysław Basak, Wiesław Wiktor Jędrzejczak

**Affiliations:** 1 Department of Hematology, Transplantation and Internal Medicine, Medical University of Warsaw, Banacha Str 1a, 02-097 Warsaw, Poland; 2 Central Laboratory of the Medical University of Warsaw, Warsaw, Poland; 3 Division of Quantitative Finance, Faculty of Economic Sciences, University of Warsaw, Warsaw, Poland

**Keywords:** Immunoglobulin G (IgG), Secondary immunodeficiencies (SIDs), Allogeneic Hematopoietic stem cell transplantation (allo-HSCT), Subcutaneous immunoglobulins, Hypogammaglobulinemia

## Abstract

Secondary immunodeficiencies are frequently observed after allo-HSCT. The efficacy of subcutaneous IgG preparations in this population is unknown. A retrospective single-institution study involved 126 adult patients transplanted in 2012–2019 for hematological malignancies. Patients were tested every 2–3 weeks for plasma IgG concentration during the 1st year after transplantation and supplemented with facilitated subcutaneous immunoglobulin when they either had IgG concentration < 500 mg/dl or between 500 and 700 mg/dl and recurrent infection. The IgG concentration < 500 mg/dL was diagnosed in 41 patients, while 500–700 mg/dL in 25 and altogether 53 patients received IgG supplementation. The median number of IgG administrations was 2. The median time to the first IgG administration after allo-HSCT was 4.1 months, while to the next administration (if more than one was required) 53 days (prophylactic group) and 32 days (group with infections). We did not observe any significant toxicity. Two situations were associated with increased probability of meeting criteria for IgG supplementation: diagnosis of either acute lymphoblastic leukemia (ALL) or chronic lymphocytic leukemia (CLL) (83.8% versus 39.3% for other diagnosis, *p* = 0.000) and the systemic use of corticosteroids (64.2% versus 31.5% for patients without systemic corticosteroids, *p* = 0.005). Over 40% of the adult recipients may require at least incidental immunoglobulin supplementation during the first year after allo-HSCT. Low IgG concentrations are associated with inferior outcomes. The subcutaneous route of IgG administration appeared to be safe and may allow for long persistence.

**Supplementary Information:**

The online version contains supplementary material available at 10.1007/s00277-021-04649-y.

## Introduction

Despite a considerable reduction in the incidence of non-relapse mortality following allogeneic hematopoietic stem cells transplantation (allo-HSCT) in recent years, infection-related mortality remains a challenge in the post-transplantation care [1, 2]. It is related to secondary immunodeficiencies that are frequently observed and are multifactorial in etiology [3–6]. According to NCCN (National Comprehensive Cancer Network) guidelines, allo-HSCT recipients (with neutrophil recovery) who require intensive immunosuppressive therapy for graft-versus-host disease (GHVD) are an example of non-neutropenic patients at great risk for infections [7]. Humoral immunodeficiencies may result in a quantitative, qualitative, and functional shortage of immunoglobulins [3–6].

However, EMA guideline on the clinical investigation of human normal immunoglobulin for intravenous administration [8] does not specify post allo-HSCT care and broadly discusses secondary immunodeficiencies to use proven failure to produce specific antibody among others as an indication for their use. Allo-HSCT recipients during the first year after transplantation fall into this category [7].

Immunoglobulin supplementation is commonly used in allo-HSCT recipients to prevent and treat infections, but also to modulate acute GvHD. However, there is an ongoing controversy about the benefit of such therapy, dosing regimens, and treatment monitoring of the immunoglobulin supplementation [7–12]. Additionally, immunoglobulin concentrates for subcutaneous administration are being more frequently administered instead of intravenous preparations, and to our best knowledge, there is no report on this subject in the available literature. Therefore, we find it important to report our experience with subcutaneous immunoglobulins.

## Material and methods

### Study population

Patients treated at the Outpatient Transplantation Service, transplanted in the years 2012–2019 were included. Patients who died shortly after allo-HSCT, and had not been referred to the Outpatient Service, were not included.

### Allo-HSCT

Conditioning was chosen on the discretion of treating physician and depended on the underlying hematological disease. Immunosuppressive treatment was combined of calcineurin inhibitor (cyclosporin or tacrolimus) and antiproliferative drug—either short course of methotrexate or mycophenolate mofetil. All patients with unrelated or mismatched donors received anti-T cell globulin (2,5-5 mg/kg daily) for 2–3 days in conditioning regimen. Immunosuppressive therapy was discontinued after 6–8 months following allo-HSCT if GVHD did not occur. Diagnosis and grading of acute and chronic GVHD were performed based on clinical symptoms and/or biopsies according to established criteria [6]. Grading of acute GvHD was performed according to Glucksberg score, while the severity of chronic GvHD-according to National Institutes of Health (NIH) Consensus Criteria 2014 [13–15]. All patients received anti-infective prophylaxis and vaccinations according to the NIH guidelines [9, 16] including prophylactic bacterial treatment with oral phenoxymethylpenicillin (1500 units twice daily) or oral levofloxacin (500-1000 mg twice daily), as described in the Supplement.

### Doses and types of immunoglobulin, the indications for supplementation therapy

All patients were systematically tested (once every 2–3 weeks) and immunoglobulin concentration was determined. Serum IgG was analyzed by the immunoturbidimetric method (Roche biochemical analyzer Cobas 8000, reagent Tina-quant IgG Gen.2) at the Central Laboratory of the Medical University of Warsaw (reference normal level: 700–1600 mg/L).

The patients received immunoglobulin supplementation based on the CDC (Centers for Disease Control and Prevention) recommendation and the NIH guidelines [6, 9]. Due to the difficulty in obtaining intravenous preparations, subcutaneous preparations were mostly used. Since the vast majority of patients had transient immunoglobulin deficiencies, IgG was administered solely if the pre-administration IgG concentration was beyond the predefined threshold.

The doses and frequencies of immunoglobulin supplementation were given according to the individual patients’ requirements. Patients with IgG level below 500 mg/dL were classified as significantly deficient and received prophylactic immunoglobulins at a dose of 0.4–0.8 g/kg. Patients with episodes of severe infection within the first 12 months after transplantation who had IgG level below 700 mg/dL received IgG supplementation at a dose of 0.5–1.0 g/kg (divided into 2 doses). Preparations used were as follows: immunoglobulin facilitated subcutaneously by recombinant human hyaluronidase (10% infusion)—HyQvia (Baxalta Innovations GmbH, Austria) or/and solution 165 mg/mL—Gammanorm (Octapharma, UK). The patients who had received intravenous IgG (IVIg) at the beginning of the study were switched to subcutaneous preparations of immunoglobulin (after at least 28 days of the last IVIg dose). Subcutaneous preparations were always administered under the abdominal skin using a variable rate portable pump according to the producer instructions. Immunoglobulin administration was preceded by hyaluronidase administration in case of HyQvia.

Adverse events were graded according to CTCAE (Common Terminology Criteria for Adverse Events v5.0) [17].

### Statistical analysis

This is a retrospective analysis. Medians, means, and standard deviations were calculated for continuous and ordered variables. In the search of predictors that increase the need for IgG supplementation, the logistic regression model was used. The following variables were tested: gender, diagnosis being an indication for transplantation, type of conditioning, type of donor, GvHD occurrence (acute and chronic GVHD were considered only if they were documented prior to the landmark of the first IgG supplementation), age of the recipient on the day of allo-HSCT, age of the recipient at the time of diagnosis, use of corticosteroids before the first IgG supplementation. Likelihood ratio test was calculated and presented along with standard error. If not mentioned otherwise, the level of statistical significance was set up at 5%. The analyses were conducted using R version 3.6.1. All time-to-event endpoints were computed from the day of allo-HSCT. The Kaplan–Meier estimator and log-rank test were used for overall survival. For the multiple comparisons, after Bonferroni adjustment for 6 comparisons between the 4 different clinical groups (A-no IgG supplementation group, B-prophylactic IgG group, C-IgG < 500 mg/dL and infection, D-IgG 500–700 mg/dL and infection), it was necessary to recalculate p[0.05/6 (6 comparisons) = 0.008] and *p* < 0.008 corresponded to the same overall level of significance.

## Results

### Patients

One hundred twenty-six patients, 74(58%) males, with median age at allo-HSCT 46 years (range 18–71) were included into the analysis. For twelve patients, it was the second allo-HSCT. The most prevalent diagnoses were acute leukemia, both myeloid (AML)-54%, and lymphoblastic (ALL)-13.5%. Baseline patients’ characteristics are shown in Table 1.

**Table 1:** Baseline patients’ characteristics (*AA* aplastic anemia, *AML* acute myeloid leukemia, *ALL* acute lymphoblastic leukemia, *GVHD* graft versus host disease, *MAC* myeloablative conditioning, *MDS* myelodysplastic syndrome, *MPN* myeloproliferative neoplasm, *MMUD* mismatched unrelated donor, *MRD* matched related donor, *MUD* matched unrelated donor, *NMA* non-myeloablative conditioning, *RIC* reduced intensity conditioning)

	Number of patients	Share of the total (%)
Gender		
Male	74	59
Female	52	41
Age in years		
18–40	49	39
40–60	55	44
> = 60	22	17
Diagnosis		
AML	68	54
MDS	13	10
ALL	17	14
AA	6	5
MPN	9	7
Lymphoma	13	10
Conditioning		
MAC	86	68
NMA	5	4
RIC	35	28
Donor		
MRD	37	30
MUD	66	52
MMUD	19	15
haplo	4	3
Acute GvHD		
Grade 1–2	28	22
Grade 3–4	11	9
Chronic GvHD		
Mild	13	10
Moderate	46	37
Severe	22	17

### Transplantations

HLA-identical siblings were used for 38(30.2%) patients, matched unrelated donors for 66(52.4%), mismatched unrelated donors for 19(15.1%), and haploidentical related donors for 3(2.4%). There were 86(68,2%) patients, who received myeloablative conditioning (MAC), 35(27.8%) reduced-intensity conditioning (RIC), and 5(4%) non-myeloablative conditioning (NMA).

### Treatment after transplantation

Thirty-four patients (27%) received additional therapy after transplantation (mainly azacitidine) aimed at preventing relapse. The detailed information on this treatment is provided in the Supplement.

### Immunosuppressive therapy

All patients received prophylactic anti-GvHD treatment, including 84 patients (67%)-cyclosporine A(CsA) and 28(22%)-tacrolimus (TAC), together with short course of methotrexate. There were 4 patients who received post-transplant cyclophosphamide (PT-Cy) after haploidentical HSCT. Other protocols included: CsA or TAC combined with mycophenolate mofetil (MMF) (4 patients(3%)) or TAC with sirolimus (6 patients(5%)). Three patients received either MMF (2 patients(2%)) or sirolimus (1 patient(1%)).

### GvHD

Acute graft-versus-host disease (aGvHD) was diagnosed in 39 patients (30.9%) including 28(22.2%) with grade 1–2, and 11(8.7%) with grade 3–4. Chronic graft-versus-host disease (cGvHD) was diagnosed in 81 patients (64%), including 13(10%) with mild, 46(37%)-moderate, and 22(17%)-severe cGvHD.

### Prevalence of IgG deficiencies after allo-HSCT

Hypogammaglobulinemia below 500 mg/dL detected at least once occurred in 32.5% of patients (41 out of 126). In most patients, hypogammaglobulinemia was occasional and transient, and only in 10.3% of patients (13 out of 126), it was chronic. Additionally, in 25 patients IgG level below 700 mg/dL but above 500 mg/dL was observed making the total number of IgG deficient patients 66 out of 126(52.4%). 47% of patients did not have a decreased IgG level and about 4% of them had hypergammaglobulinemia. Eight patients developed monoclonal gammopathy. The lowest IgG level that has served as an indication for IgG supplementation in all analyzed patients are summarized in Table 2.

**Table 2:** The lowest IgG level that has served as an indication for IgG supplementation in all analyzed patients

IgG (mg/dL)	Number of patients (Share of the total)	Prophylactic IgG (Share of the total)	Therapeutic IgG (Share of the total)	Relapse (Share of the total)	Death (Share of the total)
< 200	1 (0,1%)	1 (0,1%)	0	0	0
200–300	8 (6.3%)	4 (3.1%)	4 (3.1%)	1 (0.1%)	1 (0.1%)
300–400	13 (10.3%)	10 (7.9%)	3 (2.4%)	3 (2.4%)	3 (2.4%)
400–500	19 (15%)	10 (7.9%)	9 (7%)	4 (3.1%)	5 (4.4%)
500–700	25 (20%)	0	12 (9.4%)	3 (2.4%)	4 (3.1%)
700–1600	55 (44%)	0	0	6 (4.9%)	6 (4.9%)
> 1600	5 (4.3%)	0	0	1 (0.1%)	1 (0.1%)
Total	126 (100%)	25 (19%)	28 (22%)	18 (13%)	20 (15%)

Altogether 53 out of 126 patients (42%) received IgG supplementation with exogenous immunoglobulin. Immunoglobulin facilitated subcutaneously by recombinant human hyaluronidase was used in 85% cases and solution 165 mg/mL- in 15% cases.

IgG was administered to all patients in whom IgG level was below 500 mg/dL (41 patients (32.5%)). In 25 of them, it was only prophylactic, while in the remaining 16 also therapeutic because of recurrent infections. Additionally, 12 patients with IgG in the range 500-700 mg/dL with accompanying severe and recurrent infections also received IgG supplementation. Therefore, the entire group of patients with infection receiving IgG supplementation comprised 28 patients (16 with IgG below 500 mg/dL and 12 with IgG in the range 500-700 mg/dL). The median number of IgG administrations was 3.5 (range 1–8) in the prophylactic, and 2 (range 1–8) in the group with infection.

The occurrence of the following invasive or life-threatening infections was used as an indication for IgG administration: fungal pneumonia—7 patients (25%), bacterial pneumonia—2(7%), sepsis within preceding months—15(53%), recurrent local infection—7(24%), viral infection—25(89%) (including BK virus infection of the urinary tract—10(35.7%), CMV reactivation—10 (35.7%)), and polymicrobial infections—13 patients(46%).

Beside IgG supplementation, they received appropriate routine antimycotic, antibacterial or/and antiviral treatment. Patients with BK virus infection did not receive any antiviral treatment.

The median time to the first IgG administration was 4.1 months (range 1.1–74.2) after allo-HSCT, and 13 months after diagnosis (for the entire group) (range 4.1–173.9).

Median IgG levels before IgG therapy initiation were 460 mg/dL (range 190–480) in the prophylaxis group, 446 mg/dL (range 200–494) in patients with IgG level below 500 mg/dL, and recurrent infections and 557 mg/dL (range 519–670) in the group of patients with infections and IgG level below 700 but above 500 mg/dL.

Thirteen of 126 (10.3%) patients (3 out of 3 with chronic lymphocytic leukemia (CLL), 1 out of 4 with Hodgkin lymphoma (HL), 6 out of 68 with AML and 3 out of 17 with ALL) required regular IgG supplementation throughout the follow-up period with median 8 administrations per year (range 8–9).

While it is a retrospective and not a prospective study aiming at assessing regular pharmacokinetics, patients were controlled for IgG concentration at various time points after IgG subcutaneous administration. Nevertheless, subcutaneous IgG significantly increased IgG concentration that was maintained for 6–8 weeks. Patients originally suffering from ALL (82%) and lymphoma (58%) most frequently met the criteria for IgG supplementation and were accordingly treated. The characteristics of patients treated with IgG are shown in Table 3.

**Table 3:** Characteristics of patients treated with IgG

	Patients requiring IgG/ All patients *N* = 53/126	The proportion in total
Sex		
M	37/74	50%
F	16/52	30%
Diagnosis		
AML	20/68	29%
MDS	6/13	46%
ALL	14/17	82%
AA	2/6	33%
MPN	4/9	44%
Lymphoma	7/12	58%
(including CLL)	(3/3)	(100%)
Dono		
haplo	2/4	50%
MMUD	11/19	57%
MRD	17/37	46%
MUD	23/66	35%
Conditioning		
MAC	38/86	44%
NMA	2/5	40%
RIC	13/35	37%
GvHD		
AcuteGvHD	27/39	69%
ChronicGvHD	46/81	56%

### Survival analysis

The 1-year overall survival (1-y OS) was 97.1%(95% CI, 89.1–99.3) in the group that never required immunoglobulin supplementation, 95.8%(95% CI, 73.9–99.4) in the prophylactic IgG group, 80.6%(95% CI, 51–93.3) in the group with recurrent infections treated with IgG because of IgG level below 500 mg/dL, and finally 63.5%(95% CI, 28.9–84.7) in the group treated with IgG because of recurrent infections and IgG 500–700 mg/dL. Relapse was the primary cause of death in 7 out of 7 patients, 4 out of 5 patients, 4 out of 4 patients and 3 out of 4 patients respectively.

**Fig. 1:**
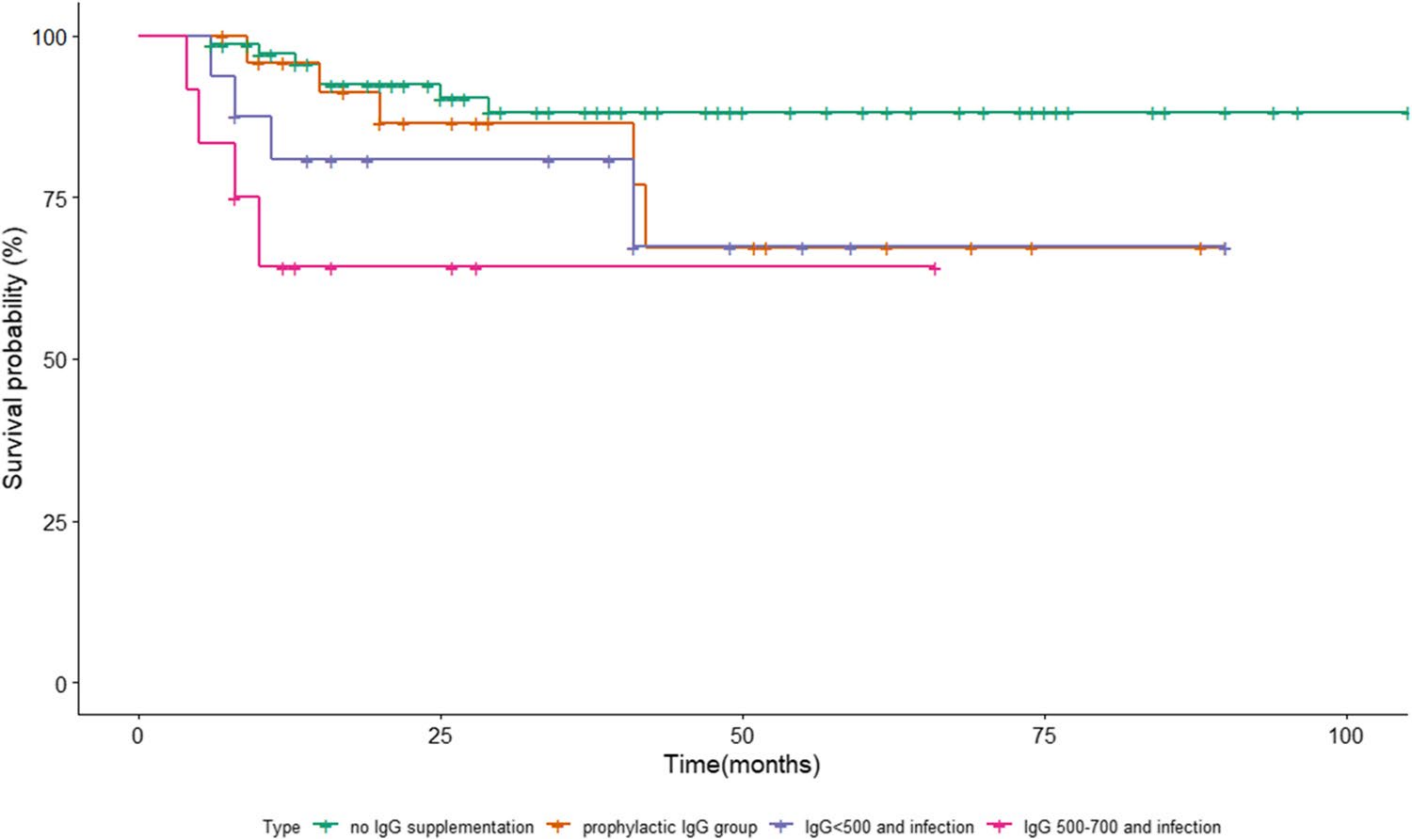
Overall survival of patients with and without IgG supplementation

The 1-y OS differences for four clinical groups (A—no IgG supplementation group, B—prophylactic IgG group, C—IgG < 500 mg/dL and infection, D—IgG 500-700 mg/dL, and infection) A vs. B, A vs. C, A vs. D, B vs. C, B vs. D, C vs. D were not statistically significant. The survival curves for all groups using Kaplan-Meier estimator are presented in Fig. 1. Detailed results of the statistically significant 1-y OS differences are shown in Table S4 (supplement).

Despite the fact that relapse contributed to death of 90% of all deceased patients, various infections complicated the course of relapse (mainly pneumonia or sepsis). Nine out of 10 patients (90%) with BKV infection receiving IgG improved, and thus achieved successful control of hemorrhagic cystitis, while 1 patient died (10%) due to relapse of the underlying disease. Detailed causes of death and diagnosed infections that occurred during the last 14 days of patients' lives are presented in Table S5 (supplement).

### Factors predictive for IgG supportive treatment

In the logistic regression model, the diagnosis of ALL or CLL and the use of systemic corticosteroid therapy were associated with the need for IgG supplementation. The average probability that a patient diagnosed with ALL or CLL will require IgG supplementation was 83.8%, while for patients with other diagnoses the average probability was 39.3% (*p* = 0.0001). The use of corticosteroids was associated with the average probability of hypogammaglobulinemia requiring IgG supplementation in 64.2%, while no corticosteroid use with 31.5% (*p* = 0.005) only.

### Toxicity

Adverse events were observed in 53% patients treated with fscIgG, including 49%—grade 1 and 4%—grade 2. In two male patients (5%) transient scrotal edema was observed, which resolved without any medical intervention. Malaise was reported in 2 patients (4%) and discomfort at the injection site in 10 patients (22%) patients. In 6 patients (13%) who suffered from chronic kidney disease—a clinically insignificant transient decrease in creatinine clearance was observed. In 3 patients (6%) of all treated patients, the change of the type of IgG products was required due to an allergic skin reaction at the application site.

## Discussion

Apart from relapse of the underlying malignancy and graft versus host disease, secondary immunodeficiency is the most common complication of allo-HSCT. All patients are affected to some degree. It results in the occurrence of infectious complications, but also in an increased risk of relapse by altered effectiveness of graft-versus-leukemia reaction [3–6, 18]. The occurrence of immunodeficiency, due to the altered and insufficient recovery of the immune system, is influenced by many factors including pre-transplant, like patient’s age, type of the underlying medical condition and its treatment (including a growing number of therapeutics targeting B cells), peri-transplant factors related to the transplantation procedure, e.g. the type of the donor, type of conditioning, ATG use and post-transplant factors i.e. multi-stage course of reconstitution, complications especially GvHD, immunosuppressive treatment and preventive treatment used after transplantation [18–22].

While this immunodeficiency is complex involving various parts and mechanisms of the immune system with limited possibility for intervention, the deficiency of immunoglobulins, particularly IgG can be corrected with exogenous preparations. Nevertheless, expert opinions regarding IgG supplementation after allo-HSCT remain ambiguous [23–27].

In our group allo-HSCT recipients, IgG deficiency was usually transient and the supplementation of IgG was interventional in most cases. Approximately 32% of recipients required incidental IgG supplementation while only 10% of them required regular supplementation every 3 weeks to maintain IgG level above 500 mg/dl. Patients received immunoglobulins when they met the predefined criteria, i.e., when either the IgG level was below 500 mg/dL, or below 700 mg/dL (but above 500 mg/dL) with frequent infections or both.

The IgG level used in this study as the indication for preventive IgG supplementation was higher than the one (< 400 mg/dL) recommended by the ASBMT (American Society for Blood and Marrow Transplantation) and EBMT (The European Society for Blood and Marrow Transplantation) guidelines [6, 9]. The reason for this modification was the use of subcutaneous preparation of IgG, which efficacy in this population of patients was unknown.

Given possible qualitative and functional shortages of immunoglobulins, we used IgG replacement as part of supportive care, for patients with higher IgG concentration who suffered with infections. The effectiveness of IgG is difficult to assess reliably because, usually, we administered causal treatment at the same time as the IgG replacement. It is worth noting that 90% of supplemented patients, suffering from symptomatic BK Polyomavirus- associated hemorrhagic cystitis in grade II or III (according to the ECIL-6 Guidelines) [28, 29] achieved the resolution of symptoms without any antiviral treatment.

The median time to the first IgG administration (4.1 months) in our analysis appears to be consistent with the course of B-cell reconstitution, described in the literature [4, 5]. Most of supplemented patients suffered from GvHD. Both acute and chronic GvHD are associated with delayed B cell reconstitution, reduction or lack of B cell precursors in the bone marrow and delays in IgG2 and IgG4 production [5, 30, 31]. Whether IgG supplementation may be useful to correct deficiencies of different IgG subclasses was beyond the scope of this study.

Cordonnier et al. reported an increased risk of fatal veno-occlusive disease/sinusoidal obstruction syndrome (VOD/SOS) in patients with acute GvHD receiving IgG replacement [32]. In our work, the shortest time to first administration was 34 days and we did not observe this complication.

Survival analysis revealed inferior survival in the group with infections than the prophylactic group but the vast majority of patients who eventually died, succumbed primarily to relapse of their leukemia with infection being only an accompanying condition. It needs further research to assess whether groups of patients with lower levels of IgG relapse more frequently than patients without IgG immunodeficiency.

In our study, the occurrence of IgG deficiency was found to be mainly dependent on the disease being an indication for transplantation, with ALL and CLL being the most common, and on systemic use of corticosteroid throughout the treatment process. In patients with B-cell malignancies total and/or functional hypogammaglobulinemia results from disease-related effects on the immune system and from side effects of the treatment [19, 26, 27]. Anti-B-cell therapy used before transplantation may be responsible for the development of post-transplantation complications. Low pre-allo-HSCT IgG level is a significant risk factor for hypogammaglobulinemia after transplantation [18–21].

In our study, no correlation for IgG replacement was found for GvHD (acute and/or chronic). The results of our analysis may be due to small number of analyzed patients, but also may indicate a stronger impact of steroid therapy than the diagnosis of GvHD on the occurrence of hypogammaglobulinemia. Corticosteroids are the current gold- standard for the treatment of GvHD [6]. According to published studies [5, 31] it is difficult to determine the causality of antibody deficiency following allo-HSCT in a situation where both GvHD and the corticosteroid therapy can lead to antibody deficiency. Greinix et al. described that impairment of reconstitution, observed in patients with GvHD (either acute or chronic) cannot be separated from the possible influence of corticosteroids [33].

To our best knowledge, this study is the first to describe the effectiveness and safety of subcutaneous preparations instead of intravenous. This treatment was well tolerated, and we did not observe any significant toxicity. According to the literature, IgG has a half-life of about 21 to 29 days following intravenous administration [34]. However, especially in patients with immunodeficiencies, interindividual variation has been reported. Taking into consideration variability of the patient's situation (different time after transplantation, different initial diagnosis, the severity of GVHD) and also the fact that they had endogenous IgG production (albeit reduced), these data have shown that subcutaneous IgG significantly increased IgG level that was maintained for 6–8 weeks.

While there was no systematic pharmacokinetic experiment, our data collected from patients more frequently tested may unexpectedly suggest that the half-life of subcutaneously administered IgG is longer than intravenously, at least in this setting, in which some endogenous IgG production is present [34–36]. This would require verification in a prospective study but is interesting and potentially useful. Promising results of the scIgG treatment may grant a possibility of at-home treatment for selected patients after allo-HSCT.

To conclude, over 40% of the adult stem cell recipients may require immunoglobulin supplementation. A vulnerable group of patients to the development of hypogammaglobulinemia after allo-HSCT are patients with B-cell neoplasms, such as acute lymphoblastic leukemia or chronic lymphocytic leukemia, as well as patients treated with corticosteroids after transplantation. Subcutaneous immunoglobulins replacement seems to be a safe and efficacious alternative to intravenous IgG preparations in patients after allo-HSCT.

## Supplementary Information

Below is the link to the electronic supplementary material.

Supplementary file1 (DOCX 20 KB)

## Data Availability

All patient data was collected based on available medical records. All of the data was analyzed anonymously.
