# Supplementary material for: Prevalence of hypogammaglobulinemia and its management with subcutaneous immunoglobulin supplementation in patients after allogeneic hematopoietic stem cell transplantation—a single-center analysis
Source: Ann Hematol. 2021 Sep 3;100(12):3007–16. doi: 10.1007/s00277-021-04649-y (PMC8593025; doi:10.1007/s00277-021-04649-y)
Supplement: Supplementary file 1 — Supplementary file1 (DOCX 20 KB) [file 277_2021_4649_MOESM1_ESM.docx]

**Supplement**

***Supportive care***

All patients received prophylactic bacterial treatment with oral phenoxymethylpenicillin (1500 units twice daily) or oral levofloxacin (500-1000mg twice daily).

Fluconazole (200-400mg daily) for patients at standard risk and posaconazole (200mg 3 times a day) or voriconazole (400mg/daily) for patients at high risk for fungal infection were used. Patients received oral acyclovir prophylaxis (800mg twice daily).

Trimethoprim–sulfametoxazole was administered as prophylaxis against Pneumocystis jiroveci infection for at least six months after engraftment.

Empirical antibiotic treatment was adjusted according to the etiology and susceptibility established in cultures.

Blood samples were tested weekly for cytomegalovirus (CMV) reactivation for the first 4-6 months after allo-HSCT and then at least once a month. The test was done using a quantitative real-time PCR for the detection of CMV-DNA in peripheral blood. In preemptive or prophylactic therapy of CMV reactivation valganciclovir was administered at a dose of 450-900mg twice daily (dependent on creatinine clearance).

The patients have been vaccinated according to schedule used for allogeneic stem cell recipients.

***Treatment after transplantation***

Seven patients (5.5%) received rituximab after allo-HSCT (4 doses: 375mg/m^2^/week for 4 consecutive weeks) because of: pure red cell aplasia after major ABO- mismatched transplantation- 4 patients (2.5%), GvHD- 1 patient (0,7%), relapse of CLL (rituximab in combination with ibrutinib)- 1 patient (0,7%), and EBV reactivation- (only two doses of rituximab)- 1 patient (0,7%). All but one patient with AML received rituximab before 2018 (years: 2015-2017).

Two patients, who suffered from CLL, were treated by ibrutinib throughout the entire studied period, due to minimal residual disease detected by cytogenetic testing.

Four patients (3%), who suffered from AML received FLT3 inhibitor (sorafenib- 3 patients (2%), gilertinib- 1 patient (0,7%), and four patients (3%), who suffered from ALL Ph(+)- dasatinib, as consolidation therapy after transplantation.

One patient (with ALL) was treated with blinatumomab because of minimal residual disease after transplantation. Another patient with partial remission of Hodgkin lymphoma was treated with brentuximab vedotin.

One of the patients with DLBCL was treated with venetoclax in combination with ibrutinib, because of primary refractory disease (succumbed after 7 months of treatment).

Fourteen patients (11%) with AML or MDS were receiving azacitidine to prevent or treat relapse, including 3 patients (2%), who additionally received donor lymphocyte infusion.

**Table S4.** Results of the statistical analysis of 1-y OS differences for all analyzed groups (A- no IgG supplementation group, B- prophylactic IgG group, C-IgG<500mg/dL and infection, D- IgG 500-700 mg/dL and infection)

|  | **p-value** |
| --- | --- |
| **A vs B** | 0.18 |
| **A vs C** | 0.009 |
| **A vs D** | 0.09 |
| **B vs C** | 0.12 |
| **B vs D** | 0.61 |
| **C vs D** | 0.35 |

**Table S5.** Causes of patients’ death and types of diagnosed active infections

|  | **Bacterial infection** | **Fungal infection** | **Viral infection** | **Causes of death** |
| --- | --- | --- | --- | --- |
| **Patient #1** | Septic shock E. coli ESBL(+) | pneumonia |  | relapse, infection, hemorrhagic stroke |
| **Patient #2** | Pneumonia, septic shock P. aeruginosa VIM |  | CMV | relapse, infection |
| **Patient #3** | Pneumonia, Clostridioides difficile inf. | pneumonia | adenoviral gastrointestinal infection | relapse, GvHD, infection, ischemic stroke |
| **Patient #4** | Pneumonia, septic shock | pneumonia | Parvo B19 | relapse, infection |
| **Patient #5** | Arthritis |  |  | relapse, infection, stroke |
| **Patient #6** | atypical pneumonia, CL. Difficile inf. |  | BKV, CMV, EBV, | Relapse, GvHD, infection |
| **Patient #7** | No data | No data | No data | relapse |
| **Patient #8** | Septic shock |  |  | GvHD, infection |
| **Patient #9** | Pneumonia | pneumonia |  | relapse, infection |
| **Patient #10** | Bacteraemia Ent. faecalis, Staph. haemolyticus | pneumonia | CMV | relapse, infection heart arrhythmia |
| **Patient #11** | No data | No data | No data | relapse, GvHD |
| **Patient #12** |  |  |  | relapse, GvHD, ARDS, pneumonia of unknown etiology |
| **Patient #13** |  |  |  | relapse, sudden death in sleep |
| **Patient #14** | No data | No data | No data | relapse |
| **Patient #15** | Pneumonia, pulmonary abscesses, Septic shock (E. coli and Ent. faecium spp. VRE) | Pneumonia | BKV, CMV | relapse, GvHD, infection |
| **Patient #16** | myositis, pneumonia, bacteraemia Kl. pneumoniae and Staph.haemoliticus | pneumonia | HCV | relapse, infection. heart arrhythmia |
| **Patient #17** |  |  |  | secondary lung cancer, GvHD |
| **Patient #18** | (unproven) Pneumonia | (unproven) Pneumonia |  | relapse, infection |
| **Patient #19** |  |  |  | relapse, cardiac death |
| **Patient #20** | No data | No data | No data | relapse |

ARDS- Acute respiratory distress syndrome
